# Immunotherapy versus chemotherapy as adjuvant therapy for resected MSI-H/dMMR colorectal cancer: real-world evidence informing precision strategies

**DOI:** 10.3389/fimmu.2025.1664684

**Published:** 2025-10-10

**Authors:** Haifeng Sun, Pin Lyu, Shuaixi Yang, Fuqi Wang, Sifan Zhai, Mengfei Zhao, Weitang Yuan, Quanbo Zhou

**Affiliations:** ^1^ Department of Colorectal Surgery, The First Affiliated Hospital of Zhengzhou University, Zhengzhou, Henan, China; ^2^ Department of Cancer Epidemiology, The Affiliated Cancer Hospital of Zhengzhou University & Henan Cancer Hospital, Zhengzhou, China

**Keywords:** colorectal cancer, microsatellite instability high, mismatch repair deficient, immunotherapy, adjuvant therapy

## Abstract

**Background​​:**

Immunotherapy has demonstrated unique advantages in MSI-H/dMMR colorectal cancer (CRC) for its later-line, first-line in metastatic status, and neoadjuvant therapy. However, evidence regarding its value in postoperative adjuvant therapy remains limited.

**Methods​​:**

We retrospectively analyzed 261 stage II/III MSI-H/dMMR CRC patients who underwent radical resection with over 2 years of follow-up. Disease-free survival (DFS) curves were used to compare prognoses under different postoperative strategies, and factors associated with recurrence were investigated.

**Results​​:**

The patients cohort (mean age 55.3, range 19-86 years and male for 56.3%) had a median follow-up of 30 (range 24-45) months. Recurrence occurred in 18 patients (6.9%), with an overall DFS rate of 93.1% during follow-up period. Overall, postoperative immunotherapy showed non-significant DFS advantage over watchful waiting (WW) (HR = 0.19, 95%CI: 0.03-1.39, P = 0.101), but it demonstrated statistically superior DFS compared to chemotherapy (HR = 0.26, 95%CI: 0.08-0.89, P = 0.033). Subgroup analyses revealed: 1) For patients achieving pathologic complete response after neoadjuvant therapy, postoperative WW and immunotherapy were equivalent (both DFS 100%); 2) For Stage II, WW and immunotherapy showed comparable DFS (HR = 0.21, 95%CI: 0.003-13.04, P = 0.463). 3) For Stage III, immunotherapy showed a trend toward superior DFS versus chemotherapy, though statistical significance was not reached (HR = 0.28, 95%CI: 0.04-1.96, P = 0.204), and both outperformed WW (HR = 0.05, 95%CI: 0.004-0.54, P = 0.014 and HR = 0.34, 95%CI: 0.05-2.17, P = 0.113, respectively). Factors significantly associated with recurrence included Lynch-negative (P = 0.02) and perineural invasion (P = 0.014).

**Conclusions​​:**

MSI-H/dMMR CRC exhibits excellent prognosis after radical surgery. Postoperative WW remains the preferred strategy for Stage II patients, While patients with stage III requires intensive adjuvant therapy, and immunotherapy may surpass conventional chemotherapy recommended by the current guidelines.

## 1 Introduction

Colorectal cancer (CRC) characterized by high microsatellite instability (MSI-H) or deficient mismatch repair (dMMR) arises from defective DNA repair machinery, leading to hypermutation and abundant neoantigen generation, which fosters a highly immunogenic microenvironment enriched with tumor-infiltrating lymphocytes and elevated expression of immune checkpoints (e.g., PD-1/PD-L1) (1). This biological uniqueness underpins their exceptional responsiveness to immune checkpoint inhibitors (ICIs) (2, 3). In 2015, Le DT et al. first discovered that MSI-H/dMMR metastatic CRC (mCRC) could significantly benefit from immunotherapy with pembrolizumab, an ICI named PD-1 monoclonal antibody. Thereafter, immunotherapy enters the later-line treatment of mCRC (4, 5). Landmark trials (KEYNOTE-177, ChecKMate-142) have established immunotherapy as first-line standard for MSI-H/dMMR mCRC, achieving objective response rates (ORR) of 45-69% and durable survival benefits (5–9). Compared to chemotherapy, the CheckMate 8HW trial recently established a huge superiority of progression-free survival in nivolumab plus ipilimumab group in MSI-H/dMMR mCRC (HR 0.21, 95% CI 0.14–0.31) (10). More recently, neoadjuvant immunotherapy has demonstrated unprecedented efficacy in both locally advanced MSI-H/dMMR rectal (11–13) and colon cancer (14, 15), potentially enabling organ preservation. The success of ICIs in advanced stages has ignited interest in extending their application to the adjuvant setting.

ICI has been reported as postoperative adjuvant therapy for melanoma (16), non-small cell lung cancer (17), and clear cell renal carcinoma (18). However, the role of postoperative immunotherapy following radical resection for stage II/III MSI-H/dMMR CRC remains poorly defined due to the persistent scarcity of clinical evidence, creating a research gap in this domain. Recently, the ATOMIC trial presented at ASCO 2025 has decisively complemented this knowledge gap. By adding atezolizumab to mFOLFOX6, it achieved an unprecedented 50% reduction in recurrence/death risk and nearly 10% increase of 3-year disease-free survival (DFS) rate from 76.6% to 86.4%, establishing the first ICI-based adjuvant standard in stage III dMMR colon cancer (19). However, this breakthrough excluded adjuvant ICI monotherapy, rectal patients, and stage II patients, leaving 50-80% of real-world MSI-H/dMMR CRC patients without evidence-based guidance.

These unresolved challenges highlight the urgent need for precision-oriented postoperative strategies beyond ATOMIC’s one-size-fits-all approach. Specifically, several questions remain unanswered: First, can WW remain the standard for stage II MSI-H/dMMR CRC given its favorable prognosis (20), or do subsets with high-risk features require escalation? Second, is chemotherapy mandatory for all stage III patients, or can ICI monotherapy suffice for low-risk subgroups (e.g., T3N1) to mitigate chemotoxicity? Third, whether to continue adjuvant therapy postoperatively in patients who achieve pathologic complete response (pCR) after neoadjuvant ICI, a fast-growing cohort excluded from the ATOMIC trial? Fourth, what biomarkers can predict recurrence risk in ICI-treated patients to facilitate precise risk stratification?

To bridge these gaps, we retrospectively conducted a real-world cohort study of 261 stage II/III MSI-H/dMMR CRC patients who underwent radical resection with over 2 years of postoperative follow-up to compare DFS of different postoperative strategies and analyze factors associated with postoperative recurrence, with the aim of complementing the clinical evidence for postoperative strategies for MSI-H/dMMR CRC.

## 2 Methods

### 2.1 Patients cohort​​

**Figure 1:**
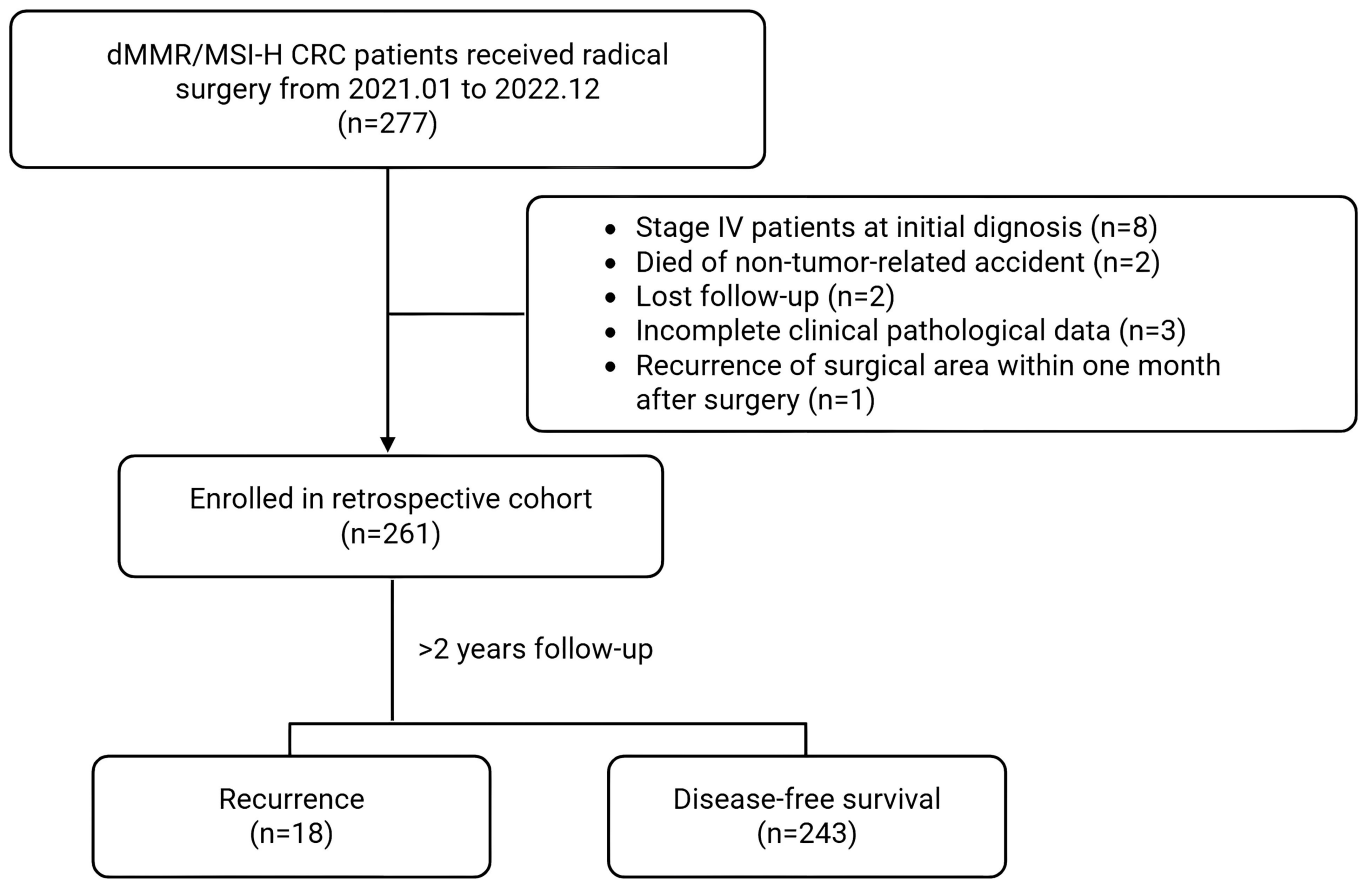
Flowchart of a study on dMMR/MSI-H CRC patients who received radical surgery from January 2021 to December 2022. Out of 277 patients, 261 were enrolled in a retrospective cohort after exclusions for various reasons. Following more than two years of follow-up, 18 experienced recurrence, while 243 achieved disease-free survival.

We conducted a real-world retrospective analysis of stage II/III MSI-H/dMMR CRC patients undergoing radical resection in the Department of Colorectal Surgery, Gastrointestinal Surgery, Gastroenterology, Oncology etc. at the First Affiliated Hospital of Zhengzhou University and Henan Cancer Hospital from January 2021 to December 2022. Inclusion criteria required: 1) CRC patients received radical resection surgery; 2) Confirmed MSI-H by PCR or next-generation sequencing, or dMMR confirmed by immunohistochemistry; 3) Postoperative pathologic stage suggestive of T3/4 with any N or N+ with any T stage (consistent pre-neoadjuvant imaging stage); 4) Over 2 years follow-up. Exclusion criteria included: 1. Stage IV (M1); 2) Death due to non-disease-related accidents during follow-up; 3) Lost to follow-up; 4) Incomplete acquisition of clinicopathological information; 5) Recurrence of the surgical area within 1 month after surgery; 6) refusal of informed consent. All patients who met the inclusion criteria were recruited strictly and consecutively to minimize selection bias. This study utilized existing clinical data without imposing additional procedures, financial burdens, or altering treatment decisions. All participants received informed consent through follow-up, with patient identities anonymized to protect privacy. The protocol was approved by the Ethics Committee of the First Affiliated Hospital of Zhengzhou University (No.2023KY0552002). Detailed in **Figure 1**.

### 2.2 Data collection​​

Complete demographic and clinicopathological information of patients cohort were collected, including gender, age, diagnosis, tumor location, histological type, clinical and pathological stage, TNM stage, whether Lynch syndrome, whether preoperative neoadjuvant therapy, postoperative pathology, postoperative strategies etc. Morning fasting blood samples collected within 24 hours of admission included: serum carcinoembryonic antigen (CEA), carbohydrate antigen 199 (CA-199), complete blood count (white blood cells [WBC], hemoglobin, neutrophils, lymphocytes, biochemical panel (albumin, pre-albumin, alanine aminotransferase [ALT], aspartate transaminase [AST], serum creatinine [Scr], and blood urea nitrogen [BUN]). We also calculated inflammatory index named CRP-albumin-lymphocyte index(CALLY) and log odds of positive nodes (LODDS), a lymph node marker calculated as log of the ratio between the number of positive nodes and the number of negative nodes, both of which were reported as prognostic predictors previously (21, 22).

### 2.3 Follow-up data

All patients were followed up after surgery according to the guidelines (23, 24), and the frequency of re-examination was once every 3 months (within 2 years after surgery) and once every 6 months (2-5 years after surgery), including tumor biomarkers, contrast-enhanced CT of chest and abdomen, contrast-enhanced MRI, and ctDNA, PET-CT, colonoscopy if necessary. DFS records the time from surgery date to recurrence or the end of follow-up. DFS curves are plotted for different patient cohorts for comparison.

### 2.4 Postoperative adjuvant therapy

Adjuvant therapy is initiated 2 to 4 weeks after surgery, with a treatment window of about 6 months. The adjuvant strategies generally follow clinical guidelines: watchful waiting (WW) for stage II, while chemotherapy based on 5-fluorouracil for stage III, unless some special circumstances, such as high-risk factors or participation in clinical trials.

The specific adjuvant therapy groups are as follows:

1. WW group (76 cases): 67 cases of stage II, while 9 cases of stage III who opted for WW due to being over 80 years old, combined with other illness including severe malnutrition, myelosuppression, and/or liver/kidney dysfunction.
2. Immunotherapy group (46 cases): 15 cases underwent ICI monotherapy, and 31 cases received ICI+chemotherapy, mainly participating in different clinical trial projects. Sintilimab and Pembrolizumab were the main used ICI drugs and have been officially approved for free use in clinical trials.
3. Chemotherapy group (139 cases): including 97 cases of stage II (combined with other risk factors and/or continuation of preoperative neoadjuvant chemotherapy) and 42 cases of stage III patients. The adjuvant chemotherapy regimen is oxaliplatin plus 5-fluorouracil, while 7 cases received 5-fluorouracil monotherapy.

### 2.5 dMMR/MSI-H testing

Immunohistochemistry (IHC) for MMR proteins (MLH1, PMS2, MSH2, MSH6), next generation sequencing (NGS), and/or PCR-based MSI testing were performed on tumor tissue samples obtained from preoperative colonoscopies or surgical resections to determine the dMMR/MSI status, and all individuals underwent at least one testing method. Among them, 232 cases underwent IHC testing, 10 cases underwent NGS, and 172 cases underwent PCR testing. 153 patients received both IHC and PCR testing, with a concordance rate of 92.2% (141 cases).

### 2.6 Statistical methods

Continuous numerical variables were presented as medians and ranges. Normally distributed continuous variables were compared using analysis of variance, non-normally distributed variables were analyzed with Mann-Whitney U test, then pairwise comparisons were conducted in multiple groups. Categorical variables were presented as numbers and percentages, and compared using the Chi-square test or Fisher’s exact test, pairwise comparisons were conducted as well. Cumulative DFS were presented using Kaplan-Meier curves, and different groups were compared using the log-rank test. Numerical and categorical variables were analyzed by independent samples t-test and chi-square test to analyze the correlation between variables and postoperative recurrence, respectively. All statistical steps were performed using SPSS 20.0 (Chicago, IL). P value < 0.05 was considered statistically significant.

## 3 Results

### 3.1 Clinicopathologic information of patients cohort​​

Among 261 patients included in the retrospective analysis, 147 (56.3%) were male and 114 (43.7%) female. The mean age was 55.3 range from 19 to 86 years old. The median follow-up time was 30 range from 24 to 45 months. During the follow-up period, no disease-related deaths occurred, and 18 patients (6.9%) experienced recurrence, with an overall DFS rate of 93.1% and the median DFS not reached. According to revised Bethesda criteria and germline mutation confirmation (25), 57 patients (21.8%) were Lynch syndrome, and dMMR occurred in 148 patients (64.6%) in MLH1-PMS2 complex, 68 (29.7%) in MSH2-MSH6 complex and 13 (5.7%) in both. Postoperative WW, ICI monotherapy, ICI+chemotherapy, and conventional chemotherapy patients are 76 (29.1%), 15 (5.7%), 31 (11.9%), 139 (53.3%) respectively. All the 18 patients with postoperative recurrence were Lynch-negative patients. See **Table 1** for details.

**Table 1:** Basic data of MSI-H/dMMR CRC patients (%).

Variables	Value	Disease-free survival	Recurrence
n	261	243	18
Mean age (range, years)	55.3 (19-86)	55.0 (19-86)	59.6 (39-80)
Median follow-up (range, months)	30 (24-45)	30 (24-45)	33 (24-39)
DFS,%	93.10	100	0
Gender			
Male	147 (56.3)	138 (93.9)	9 (6.1)
Female	114 (43.7)	105 (92.1)	9 (7.9)
Lynch Syndrome			
Yes	57 (21.8)	57 (100)	0
No	204 (78.2)	186 (91.2)	18 (8.8)
Mismatch repair-deficient			
MLH1-PMS2 complex	148 (64.6)	138 (93.2)	10 (6.8)
MSH2-MSH6 complex	68 (29.7)	63 (92.6)	5 (7.4)
Both	13 (5.7)	12 (92.3)	1 (6.9)
Tumor location			
Ascending colon	82 (31.4)	77 (93.9)	5 (6.1)
Transverse colon	53 (20.3)	52 (98.1)	1 (1.9)
Descending colon	22 (8.4)	20 (90.9)	2 (9.1)
Sigmoid colon	27 (10.3)	25 (92.6)	2 (7.4)
Rectum	62 (23.8)	56 (90.3)	6 (9.7)
Multiple tumors	15 (5.7)	13 (86.7)	2 (13.3)
Histological types			
Adenocarcinoma	179 (68.6)	163 (91.1)	16 (8.9)
Mucinous adenocarcinoma	55 (21.1)	53 (96.4)	2 (3.6)
Ring cell carcinoma	2 (0.8)	2 (100)	0
pCR	25 (9.6)	25 (100)	0
Postoperative strategies			
Watchful waiting	76 (29.1)	72 (94.7)	4 (5.3)
ICI monotherapy	15 (5.7)	15 (100)	0
ICI+chemotherapy	31 (11.9)	31 (100)	0
Chemotherapy	139 (53.3)	125 (89.9)	14 (10.1)

DFS, Post-operative disease-free survival; pCR, Pathological complete remission, ICI, Immune checkpoint inhibitor.

The DFS rates of postoperative WW, immunotherapy (with or without chemotherapy) and chemotherapy groups were 94.74%, 100.00% and 89.93%, respectively. Except for pathological type and clinical stage, there were no significant differences in gender, age, tumor markers, tumor location, dMMR type and pathological high factors among the three groups. Detailed in **Table 2**.

**Table 2:** Clinicopathological characteristics of patients grouped by different postoperative strategies(%).

Variables	Watchful waiting	Immunotherapy	Chemotherapy	P
N	76	46	139	
Gender				
Male	42 (55.3)	25 (54.3)	80 (57.6)	0.91
Female	34 (44.7)	21 (45.7)	59 (42.4)	
Age (years)	56.4 ± 15.0	52.0 ± 12.7	55.9 ± 12.3	0.16
Lynch Syndrome				0.22
Yes	13 (17.1)	14 (30.4)	30 (21.6)	
No	63 (82.9)	32 (69.6)	109 (78.4)	
CEA (ng/ml)	7.98 ± 21.53	6.20 ± 9.40	5.82 ± 19.42	0.73
CA-199 (U/ml)	22.54 ± 51.37	36.24 ± 57.52	28.50 ± 64.65	0.49
Tumor location				0.29
Ascending colon	26 (34.2)	9 (19.6)	47 (33.8)	
Transverse colon	19 (25.0)	8 (17.4)	26 (18.7)	
Descending colon	3 (3.9)	7 (15.2)	12 (8.6)	
Sigmoid colon	6 (7.9)	4 (8.7)	17 (12.2)	
Rectum	19 (25.0)	15 (32.6)	28 (20.1)	
Multiple tumors	3 (3.9)	3 (6.5)	9 (6.5)	
Histological types				<0.01*#&
Adenocarcinoma	54 (71.1)	20 (43.5)	105 (75.5)	
Mucinous adenocarcinoma	13 (17.1)	10 (21.7)	32 (23.0)	
Ring cell carcinoma	0	1 (2.2)	1 (0.7)	
pCR	9 (11.8)	15 (32.6)	1 (0.7)	
Mismatch repair-deficient				0.44
MLH1-PMS2 complex	42 (63.6)	22 (56.4)	84 (67.7)	
MSH2-MSH6 complex	20 (30.3)	16 (41.0)	32 (25.8)	
Both	4 (6.1)	1 (2.6)	8 (6.5)	
Clinical stage				0.01&
II	67 (88.2)	34 (73.9)	97 (69.8)	
III	9 (11.8)	12 (26.1)	42 (30.2)	
High risk factors				
Vascular invasion	15/76 (19.7)	10/46 (21.7)	42/139 (30.2)	0.38
Perineural invasion	15/76 (19.7)	12/46 (26.1)	51/139 (36.7)	0.14
Tumor budding	30/76 (39.5)	17/46 (37.0)	72/139 (51.8)	0.41
Recurrence, n	4	0	14	–
Local recurrence	1 (25.0)	0	2 (14.3)	
Metastasis	3 (75.0)	0	12 (85.7)	
DFS, %	94.74	100.00	89.93	–

CEA, Carcinoembryonic antigen, CA-199,Carbohydrate antigen 199, pCR, Pathological complete remission, DFS, Post-operative disease-free survival

Immunotherapy group includes ICI monotherapy (n=15) and ICI+chemotherapy (n=31)

Overall test: Chi-square test for categorical variables, and One-way ANOVA for continuous variables.

Pairwise test: Statistical differences represented as *(Immuno vs WW), #(Immuno vs Chemo), &(Chemo vs WW), respectively.

Total of 25 of 45 patients received neoadjuvant therapy achieved pCR (22/25 in neoadjuvant immunotherapy and 3/20 in neoadjuvant chemotherapy). Not shown in table.

### 3.2 Prognostic analysis of different postoperative strategies for MSI-H/dMMR colorectal cancer

All the MSI-H/dMMR CRC patients survived during the follow-up period, and the median OS and DFS were not reached. We then performed pairwise comparisons among three different postoperative strategies to identify specific differences.

#### 3.2.1 Watchful waiting vs. immunotherapy

**Figure 2:**
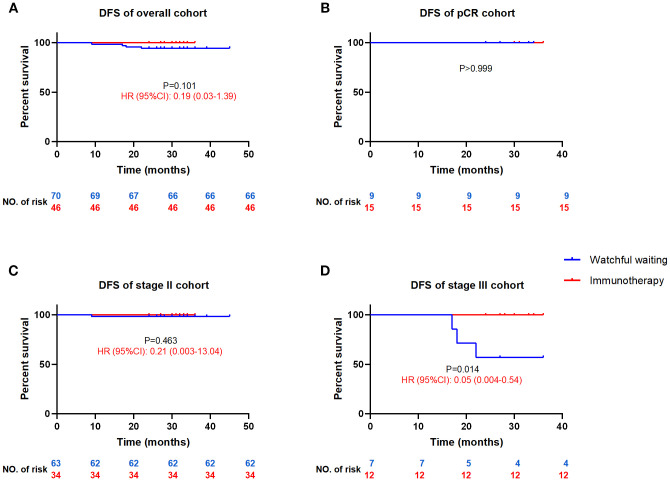
Comparison of DFS between the postoperative watchful waiting and immunotherapy for MSI-H/dMMR colorectal cancer patients DFS, disease-free survival; HR, hazard ratio (watchful waiting as reference), CI, confidence interval. DFS curves for all cohort patients **(A)**, patients achieved pCR **(B)**, stage II patients **(C)** and stage III patients **(D)**.

Postoperative immunotherapy showed superiority over WW on the DFS curve, but did not reach a statistical difference (HR = 0.19, 95%CI: 0.03-1.39, P = 0.101, **Figure 2A**). There is still a lack of clinical evidence as to whether pCR patients after neoadjuvant therapy need to continue postoperative consolidation treatment. Our results showed no postoperative recurrence of WW or immunotherapy, suggesting that WW may be the better choice for pCR patients (**Figure 2B**). According to current guidelines, MSI-H/dMMR CRC patients are recommended WW for stage II and adjuvant treatment for stage III after surgery. Subgroup analysis showed a significant DFS advantage of immunotherapy over the WW in stage III (HR = 0.05, 95%CI: 0.004-0.54, P = 0.014, **Figure 2D**) and no difference in stage II (HR = 0.21, 95%CI: 0.003-13.04, P = 0.463, **Figure 2C**) reaffirming the guideline recommendation.

#### 3.2.2 Immunotherapy vs. chemotherapy

**Figure 3:**
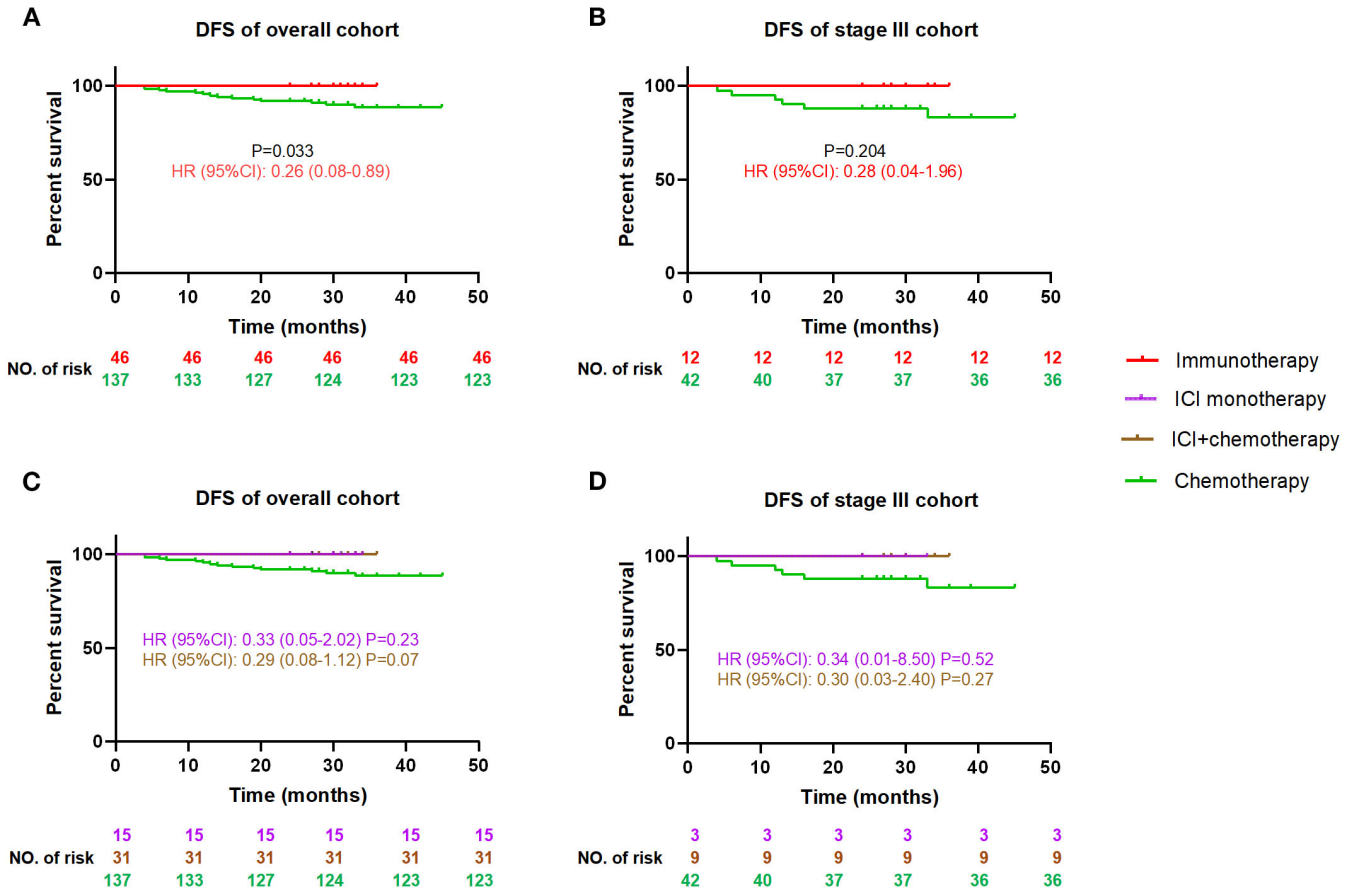
Comparison of DFS between the postoperative immunotherapy and chemotherapy for MSI-H/dMMR colorectal cancer patients DFS, disease-free survival; HR, hazard ratio (chemotherapy as reference), CI, confidence interval, ICI, immune checkpoint inhibitor. DFS curves between immunotherapy and chemotherapy for all cohort patients **(A)**, and stage III patients **(B)**; DFS curves among ICI monotherapy, ICI+chemotherapy and chemotherapy for all cohort patients **(C)**, and stage III patients **(D)**.

There is a lack of evidence for adjuvant immunotherapy after surgery for MSI-H/dMMR CRC, and guidelines only recommend conventional chemotherapy stage III. While the overall cohort demonstrated a statistically significant DFS advantage of immunotherapy over chemotherapy (HR = 0.26, 95%CI: 0.08-0.89, P = 0.033, **Figure 3A**), the stage III subgroup analysis showed a consistent magnitude of benefit (HR = 0.28, 95%CI:0.04-1.96) that did not reach statistical significance due to reduced sample size (P = 0.204, **Figure 3B**). There was no difference in DFS between ICI with and without chemotherapy, but both showed a DFS advantage over chemotherapy alone (0.29<all HR<0.34), although the statistical difference was lost due to reduced sample size (0.07<all P<0.52, **Figures 3C, D**).

#### 3.2.3 Chemotherapy vs. watchful waiting

Overall, MSI-H/dMMR CRC presents favorable prognosis. Only 9 patients with stage III in our patients cohort opted for WW instead of guideline-recommended adjuvant chemotherapy. Subgroup analysis of stage III patients revealed that, although the chemotherapy approach showed no statistically significant difference compared to WW due to small sample size (HR = 0.34, 95%CI: 0.05-2.17, P = 0.113), it demonstrated a better trend in the DFS curve (**Supplementary Figure 1**).

### 3.3 Factors associated with postoperative recurrence in MSI-H/dMMR CRC patients

To facilitate prognostic risk stratification for MSI-H/dMMR CRC patients, we analyzed the correlation between multiple clinicopathological factors and postoperative recurrence. The results demonstrated that Lynch-negative (P = 0.02) and perineural invasion (P = 0.014) were significantly correlated with recurrence, whereas no other factors showed statistical significance, as detailed in **Table 3**.

**Table 3:** Correlation factors of postoperative recurrence.

Numeric variables	T	P value	Categorical variables	Pearson χ²	P value
Age	-1.404	0.162	Gender	0.314	0.575
CEA	-1.002	0.33	Lynch syndrome	5.402	0.02
CA199	0.985	0.326	Mismatch repair-deficient	0.06	0.996
Hemoglobin	0.31	0.757	Postoperative strategies	5.906	0.116
White blood cell	1.095	0.275	CALLY	5.868	0.015
Lymphocyte count	0.245	0.807	LODDS	13.583	<0.001
Neutrophil count	0.744	0.458	Vascular invasion	1.77	0.183
NLR	0.171	0.864	Perineural invasion	6.08	0.014
Albumin	-0.388	0.698	Tumor budding	0.35	0.554
Pre-albumin	-0.149	0.882			
ALT	-0.881	0.379			
AST	-1.029	0.317			
Urea nitrogen	-1.13	0.273			
Serum creatinine	-0.992	0.322			

CEA, Carcinoembryonic antigen, CA-199, Carbohydrate antigen 199, ALT, Alanine aminotransferase, AST, Aspartate transaminase, NLR, Neutrophil-lymphocyte ratio, CALLY, CRP-albumin-lymphocyte index, LODDS, Log odds of positive lymph nodes.

Independent sample t-tests for numeric variables, Chi-square test for categorical variables.

## 4 Discussion

Our real-world study provides evidence redefining postoperative management for MSI-H/dMMR CRC. The 93.1% overall DFS rate at median 30-month follow-up corroborates the exceptional prognosis of this subtype, challenging conventional adjuvant paradigms. Most notably, we established that WW remains appropriate for stage II patients (95.5% DFS), while revealing immunotherapy’s potential superiority over chemotherapy in stage III disease (HR = 0.28, 95%CI: 0.04-1.96).These results support the latest guidelines that explicitly advise WW for stage II, but compel reconsideration of guidelines recommending blanket chemotherapy for stage III CRC regardless of MMR status (23, 26, 27) given chemotherapy’s established neurotoxicity (28) and marginal benefit in dMMR tumors (29, 30). Another finding is that patients achieving pCR after neoadjuvant ICIs had pretty good DFS (100%) in our cohort. However, given the retrospective nature and limited sample size, we still need to be cautious about the organ preservation strategy for those cCR acguevers after neoadjuvant therapy (31, 32).

The contrast between immunotherapy and chemotherapy was the main finding. While the overall cohort demonstrated a statistically significant DFS advantage of immunotherapy over chemotherapy (HR = 0.26, 95% CI 0.08–0.89; P = 0.033), the stage III subgroup analysis showed a consistent magnitude of benefit (HR = 0.28, 95% CI 0.04–1.96) but lost statistical significance (P = 0.204). This apparent discrepancy primarily stems from limited statistical power in the stage III immunotherapy cohort (n=12), reflecting the real-world constraint of off-guideline treatment adoption. Despite the small sample size, the clinically meaningful separation of Kaplan-Meier curves (**Figure 3B**) aligns with the significant effect observed in the overall analysis (**Figure 3A**), suggesting that the lack of statistical significance likely represents a type II error rather than a true absence of treatment effect.

While the practice-changing ATOMIC trial would soon establish ICI+chemotherapy as adjuvant standard for stage III dMMR colon cancer, our findings well complement its critical limitations: First, by validating WW for stage II patients (excluded in ATOMIC), we provide evidence for about 40% of real-world dMMR CRC population currently without guidance. Second, the same efficiency of ICI monotherapy and ICI+chemotherapy suggests chemotherapy may be safely omitted in select patients, particularly considering the 43.1% incidence of grade ≥3 adverse events in ATOMIC (19). This suggests that the benefit of ICI+chemotherapy in the ATOMIC trial may be primarily derived from ICI, and we call for future large prospective studies of postoperative ICI monotherapy to support treatment intensity de-escalation.

Previous reports have confirmed the advantages of immunotherapy over chemotherapy in dMMR/MSI-H CRC. For dMMR/MSI-H mCRC, the Keynote-177 study reported that ICI monotherapy significantly outperformed chemotherapy in terms of PFS, OS, and adverse events (8), while the CheckMate-8HW study found that dual ICIs was even more effective. Our comparative data focuses on non-metastatic dMMR/MSI-H CRC patients who have undergone radical surgery (10), and we draw similar conclusions of immunotherapy’s superiority over chemotherapy in the phase III subgroup. As for non-metastatic dMMR/MSI-H CRC, the NICHE series of studies has broadened the applicable scenarios for neoadjuvant immunotherapy, although there are no direct comparisons to chemotherapy (14, 15), its extremely high pCR (68%) is far superior to conventional neoadjuvant chemotherapy (<30% pCR) (33–35). Our study will provide evidence to continue to broaden the applicable scenarios for postoperative adjuvant immunotherapy.

In China, ICIs and chemotherapeutic agents are almost entirely covered by the national medical insurance system, rendering the economic burden of immunotherapy comparable to that of chemotherapy but substantially lower than targeted therapy. Although ICIs may be associated with severe immune-related adverse events (e.g., myocarditis), their overall incidence remains lower compared to adverse events induced by chemotherapy (36–39). Furthermore, immunotherapy shows better DFS and reduced neurotoxicity. Ultimately, immunotherapy demonstrates superior long-term cost-effectiveness and quality-of-life outcomes relative to chemotherapy. Formal cost-effectiveness analysis is warranted.

Our biomarker analysis reveals actionable stratification tools. Lynch syndrome has been reported to not only show a better prognosis (40, 41) but also a better response to immunotherapy (NICHE 2 trial) (14) compared with sporadic MSI-H/dMMR CRC, but negative results have also been reported (42). Despite the controversy, our results suggest that Lynch syndrome is a strong protective factor in reducing postoperative recurrence (0% vs 8.8% in sporadic cases). The possible mechanism is that Lynch-associated tumors exhibit higher neoantigen burden and T-cell infiltration (43). Current guidelines define MSI-H/dMMR as a low-risk factor regardless of the results of traditional high-risk factors, such as vascular and perineural invasion (23, 24, 27). Our results suggest that perineural invasion should still be considered as a prognostic stratification and treatment guidance factor in MSI-H/dMMR CRC patients. Lynch-negative and perineural invasion together identify patients in need of intensive therapy, while patients without high-risk markers can be considered for treatment de-escalation, such as Lynch syndrome.

This is the first real-world study comparing immunotherapy and chemotherapy as postoperative adjuvant therapy for MSI-H/dMMR CRC. However, study have limitations include retrospective design and modest recurrence events (n=18) constraining multivariate analysis. The chemotherapy cohort’s higher recurrence (10.07% vs immunotherapy’s 0%) may reflect residual confounding by high-risk feature clustering. Ongoing clinical trials will prospectively validate our framework, such as PACE trial (NCT05236972) for ICI monotherapy (44). Future research must define optimal ICI duration and integrate emerging biomarkers like ctDNA clearance, particularly for pCR patients where adjuvant omission appears safe. Which is better, preoperative neoadjuvant immunotherapy or postoperative adjuvant immunotherapy? Moreover, cost-effectiveness analyses should evaluate WW/immunotherapy against chemotherapy in resource-limited settings.

**Figure 4:**
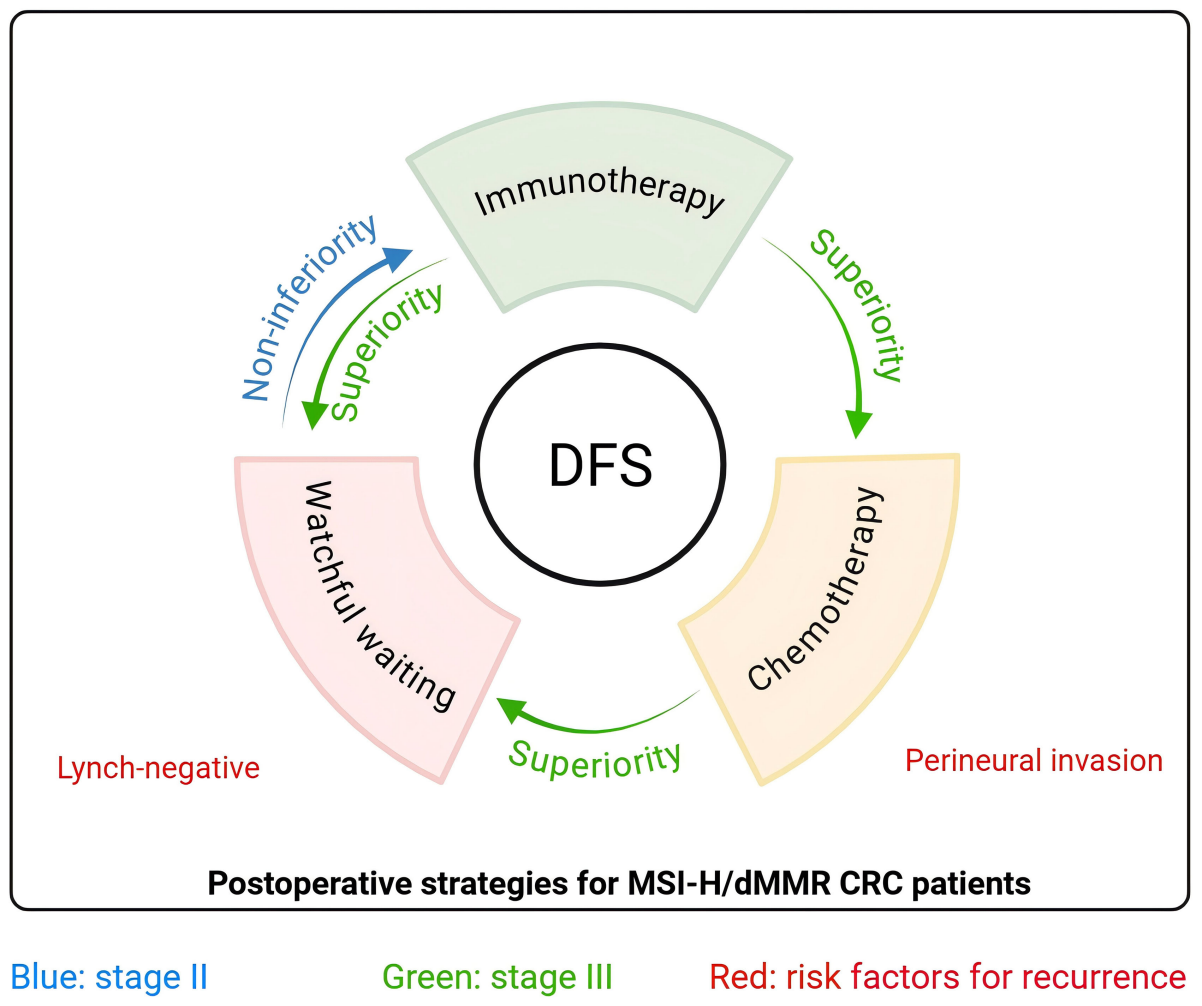
Graphical abstract Strategic framework for postoperative management of MSI-H/dMMR CRC patients. For stage II (blue arrow), watchful waiting is non-inferior to immunotherapy as the preferred strategy postoperatively. For stage III (green arrow), immunotherapy is superior to chemotherapy and watchful waiting as the preferred strategy postoperatively. Lynch-negative and perineural invasion are risk factors (red) for postoperative recurrence. DFS, disease-free survival; CRC, colorectal cancer.

Our results establish a strategic framework for postoperative management of stage II/III MSI-H/dMMR CRC (**Figure 4**). For stage II patients, WW demonstrates non-inferior DFS to immunotherapy, validating current guideline endorsements for WW strategy in this subgroup. Conversely, in stage III patients, immunotherapy may outperform guideline-recommended chemotherapy, emerging as a superior alternative for DFS optimization. We further emphasize the need to individualize treatment based on the strategic framework, by using postoperative recurrence risk factors (Lynch-negative, perineural invasion) to facilitate precise risk stratification and minimize overtreatment.

## Data Availability

The raw data supporting the conclusions of this article will be made available by the authors, without undue reservation.

## Supplementary material

Supplementary Figure 1 Comparison of DFS between the postoperative watchful waiting and chemotherapy for stage III MSI-H/dMMR colorectal cancer patients DFS, disease-free survival; HR, hazard ratio (chemotherapy as reference), CI, confidence interval.
