# Supplementary figures and images for: Immunotherapy versus chemotherapy as adjuvant therapy for resected MSI-H/dMMR colorectal cancer: real-world evidence informing precision strategies
Source: Front Immunol. 2025 Oct 10;16:1664684. doi: 10.3389/fimmu.2025.1664684 (PMC12549702; doi:10.3389/fimmu.2025.1664684)

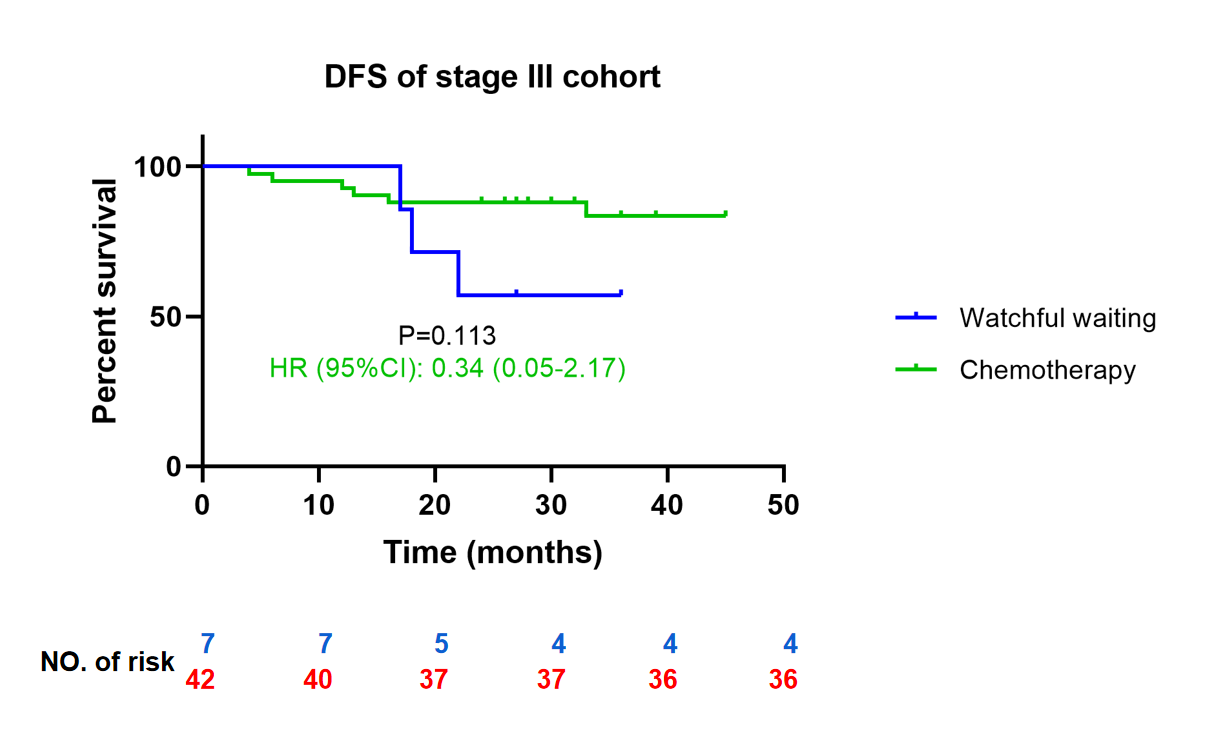

Supplement: Supplementary Figure 1 — Comparison of DFS between the postoperative watchful waiting and chemotherapy for stage III MSI-H/dMMR colorectal cancer patients DFS, disease-free survival; HR, hazard ratio (chemotherapy as reference), CI, confidence interval. [file Image1.tif]
